# Batch and Fixed-Bed Column Studies on Palladium Recovery from Acidic Solution by Modified MgSiO_3_

**DOI:** 10.3390/ijerph17249500

**Published:** 2020-12-18

**Authors:** Cosmin Vancea, Maria Mihailescu, Adina Negrea, Giannin Mosoarca, Mihaela Ciopec, Narcis Duteanu, Petru Negrea, Vasile Minzatu

**Affiliations:** 1 Faculty of Industrial Chemistry and Environmental Engineering, Politehnica University Timisoara, 300223 Timisoara, Romania; cosmin.vancea@upt.ro (C.V.); mihailescumia@gmail.com (M.M.); adina.negrea@upt.ro (A.N.); narcis.duteanu@upt.ro (N.D.); petru.negrea@upt.ro (P.N.); 2 Research Institute for Renewable Energy, Politehnica University Timisoara, 138 Musicescu Street, 300774 Timisoara, Romania; vasile.minzatu@student.upt.ro

**Keywords:** palladium recovery, MgSiO_3_, DL-cysteine, batch adsorption, fixed-bed column adsorption

## Abstract

Effective recovery of palladium ions from acidic waste solutions is important due to palladium’s intensive usage as a catalyst for different industrial processes and to the high price paid for its production from natural resources. In this paper, we test the ability of a new adsorbent, MgSiO_3_ functionalized by impregnation with DL-cysteine (cys), for palladium ion recovery from waste solutions. The Brunauer–Emmett–Teller (BET) surface area analysis, Barrett–Joyner–Halenda (BJH) pore size and volume analysis, scanning electron microscopy (SEM), energy dispersive X-ray (EDX) spectroscopy and Fourier-Transformed Infrared (FTIR) spectroscopy have been performed to characterize this material. Firstly, the maximum adsorption capacity of the new obtained material, MgSiO_3_-cys, in batch, was studied. To establish the adsorption mechanism, the obtained experimental data were fitted using the Langmuir, Freundlich and Sips adsorption isotherms. Studies on the adsorption of palladium ions on the synthesized material were performed in a dynamic regime, in a fixed-bed column. The Pd(II) recovery mechanism in the dynamic column regime was established based on Bohart–Adams, Yoon–Nelson, Thomas, and Clark models. The obtained equilibrium adsorption capacity was 9.3 (mg g^−1^) in static regime (batch) and 3 (mg g^−1^) in dynamic regime (column). The models that best describe the Pd(II) recovery process for batch and column adsorption are Sips and Clark, respectively.

## 1. Introduction

The noble metals platinum, palladium and rhodium have a wide application range based on their distinct physical and chemical properties [1,2,3,4,5]. One of the first historical uses of the precious metals was as currency, internationally recognized under ISO 4217. Palladium and its alloys are currently used by the telecommunication and automotive industries (as catalytic converters), the metallurgy and chemical industries, for jewelry manufacturing and in the medical field (dental alloy production) [5,6,7,8,9].

Its growing popularity led, in 2010, to official recognition as the fourth most precious metal, after gold, silver and platinum, a statute that requires the marking of each jewel. White gold contains variable amounts of palladium (up to 20%); furthermore, dental alloys can contain up to 10% palladium [5,10,11,12,13,14,15].

Due to its increased used, palladium has become an important contact allergen, with palladium-positive patch-tests being reported by different countries—from 2% in Northern Ireland to 13% in Israel. The main aspects of palladium allergy are contact dermatitis, rash for lichen planes, stomatitis, and mouth burns [16].

Because natural platinum resources are limited, the recovery of platinum-group metals is a challenge of great importance. The two main classes of recyclable precious metal wastes are (i) precious metals including tableware, jewelry, coins, electronic waste, and spent catalysts, and (ii) secondary products of recovery/processing/use of precious metals and, namely, waste: sludge, anodic sludge, ash, filters, refractory materials, crucibles, photographic films, and resins [5].

Palladium compounds are toxic and carcinogenic for humans. Palladium is easily accumulated into plants, transported through their roots, and, finally, along the food chain [17]. Excessive exposure to palladium has adverse effects on human health, such as skin and eye irritation, substantial DNA and cell mitochondrial degradation, and deterioration and inhibition of hydroxyl radicals associated with enzymatic activity [18]. As environmental issues related to palladium contamination continue to grow, finding advantageous extracting alternatives becomes very important. Some of the analytical techniques used for palladium ion recovery by separation and preconcentration are coprecipitation, solvent extraction, electro-deposition, or membrane filtration [19,20,21,22,23,24,25].

These techniques have some major disadvantages such as lack of sensitivity and selectivity, high cost, and high consumption of toxic organic solvents [26,27,28,29]. Recently, a new separation technique has emerged, having many advantages over traditional extraction methods: high enrichment factor, better separation, selectivity and high efficiency, ease of recovery and reuse, low cost due to the low consumption of organic reagents and solvents, and, most importantly, environmentally friendly. This method consists of using a preconcentration of the metal ion of new materials obtained by functionalization of chemically inert supports with various pendant groups. Some of the advantages of these new materials are high chemical and thermal stability, fast availability, economic viability, and easy and robust surface immobilization. The materials most commonly used as supports are commercial polymeric resins [30,31,32], resins such as Dowex 1-X10 [33], Bio-Rad AG1-8x [34], Dowex 1-X8 and Amberlite IRN -78 [35,36,37,38,39], Amberlite IRA-958, Lewatit MP-500 A, and Purolliet A-850 [40,41], natural polymers [42], inorganic silicone compounds [43], magnesium silicate [44,45], and ionic liquids crown ethers [46].

Selection of the sorbent material is a complex decision, the choice being influenced by the palladium form in the considered waste and many other factors. The absorbent materials technology can be efficient at the laboratory scale, but could be a failure at industrial level. Thus, the choice of sorbent material in close correlation with treatment technology for liquid acid waste is very important.

Adsorption is an advanced method, efficient and with high potential for metal ion removal from aqueous solutions. Therefore, it is important to develop new materials having advanced sorbent properties. Materials can be improved by functionalization of the solid support with an extractant containing certain groups designed to improve the sorbent properties of the support. Organic compounds with structural N, P, or S pendant groups can be used as extractors for functionalization of the support [44,47].

Therefore, new chemical modification methods of the inorganic or organic supports have been developed by functionalization with different extractants.

Common techniques at present include the wet method, the dry method, the adding modifiers method, and the dynamic column method, which has the following advantages: a short functionalization time and an increased effectiveness of the adsorption process [48,49,50,51,52,53].

In order to apply these methods, some restrictions must be considered—the extractant must be liquid or be kept in a liquid state by the addition of solvent; the extractant and solvent must have a minimum solubility; the support must be prepared for impregnation; and the functionalization method must not alter the properties of the extractant or support [51].

The purpose of this study was to develop an ecological strategy for Pd(II) recovery using (florisil) MgSiO_3_ functionalized by impregnation with DL-cysteine (cys) [49]. This new material has -SH, -NH_2_, and -COOH pendant groups derived from the amino acid DL-cysteine [54,55].

The first objective was to test the new MgSiO_3_ cys material’s ability to recover palladium ions from waste solutions by adsorption. The second goal of this research was to compare the static adsorption process with the dynamic one.

## 2. Materials and Methods

### 2.1. Adsorbent Synthesis and Characterization

**Figure 1:**
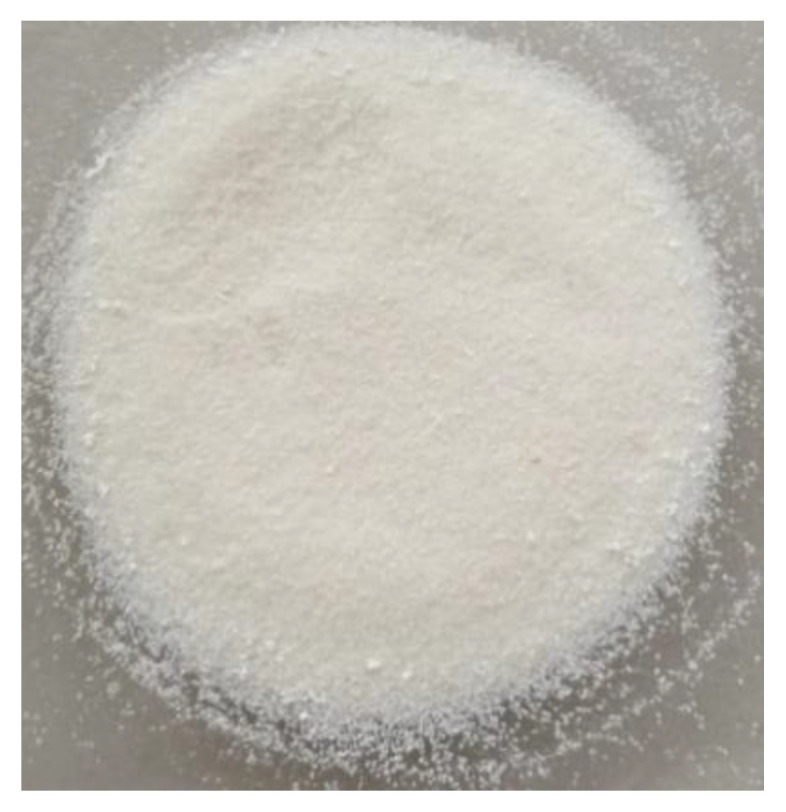
The appearance of the adsorbent material obtained by functionalizing MgSiO_3_ using DL-cysteine.

Functionalized MgSiO_3_ using DL-cysteine (DL-cysteine-hydrochloride monohydrate 99.0%, Fluka, Buchs, Switzerland) as extractant was obtained using 0.1 g of DL-cysteine. This amount of extractant was dissolved in 25 mL of deionized water. The obtained solution was mixed with 1 g of support (MgSiO_3_, 60–100 mesh, Merck, Darmstadt, Germany), corresponding to a ratio support: extractant of 1:0.1 and they were brought into contact for 24 h in stand-by (SIR, solvent impregnated resin—dry method) [49,51]. After that, the obtained material was dried in the oven (Pol-Eko SLW 53 STD, POL-EKO-APARATURA, Wodzisław Śląski, Poland) for 24 h at 323 K. The appearance of the adsorbent material can be seen in Figure 1.

The specific surface area, cumulative pore volume, and pore size of the adsorbent material were measured with a Micromeritics ASAP 2020 instrument (Brunauer–Emmett–Teller, BET, surface area analysis and Barrett–Joyner–Halenda, BJH, pore size and volume analysis, at liquid nitrogen temperature, −196 °C) from Micromeritics Instrument, Norcross, GA, USA. The point of zero charge (pH_PZC_) and density were determined using the solid addition method and pycnometer method, respectively. Furthermore, the adsorbent was analyzed by scanning electron microscopy (SEM) and energy dispersive X-ray (EDX) spectroscopy, using the FEI Quanta FEG 250 instrument (FEI, Eindhoven, The Netherlands), and Fourier-Transformed Infrared (FTIR) spectroscopy using a Bruker Platinum ATR-QL Diamond apparatus (Bruker Optik GmbH, Ettlingen, Germany) in the range of 4000–400 cm^−1^.

### 2.2. Batch Adsorption Experiments

The effect of the initial concentration of Pd(II) upon the adsorption capacity of the materials was studied using Pd(II) solutions of different concentrations (5, 10, 20, 30, 40, and 50 mg L^−1^), prepared by the appropriate dilution of a stock solution of palladium (II) chloride (5 wt% in 10 wt% HCl, Sigma-Aldrich, St. Louis, MO, USA). Adsorptions were carried out at pH = 2 for one hour at 298 K. The equilibrium concentration was determined using thermostatic Julabo SW23 water bath and shaken at a rotation speed of 200 rpm. The adsorption mechanism was established by modeling the experiment using three specific isotherms in non-linear form: Langmuir, Freundlich and Sips, according to equations used in scientific literature [56,57,58,59].

(1) $$\mathrm{Langmuir}\;q_{e}=\frac{q_{m}\cdot K_{L}\cdot C_{e}}{1+K_{L}\cdot C_{e}}$$

(2) $$\mathrm{Freundlich}\;q_{e}=K_{F}\cdot C_{e}^{1/n_{F}}$$

(3) $$\mathrm{Sips}\;q_{e}=\frac{q_{m}\cdot K_{S}\cdot C_{e}^{1/n_{S}}}{1+K_{S}\cdot C_{e}^{1/n_{S}}}$$

where *q_e_* is the maximum absorption capacity (mg g^−1^), *q_m_* is maximum adsorption capacity (mg g^−1^), *K_L_* is the Langmuir constant, *C_e_* is the equilibrium concentration of Pd(II) in solution (mg L^−1^), *K_F_* is the Freundlich constant, 1/*n_F_* is the heterogeneity factor, *K_S_* is Sips constant, and 1/*n_S_* is the Sips model exponent.

Three independent replicates were performed for each batch adsorption experiment.

### 2.3. Column Adsorption Experiments

The obtained sorbent, MgSiO_3_ functionalized using DL-cysteine (MgSiO_3_- cys), was used for dynamic studies in a fixed-bed column. Pd(II) solutions’ initial concentration was 60 (mg L^−1^), prepared using appropriate dilution of a stock solution of palladium (II) chloride (5 wt% in 10 wt% HCl, Sigma-Aldrich, St. Louis, MO, USA).

**Figure 2:**
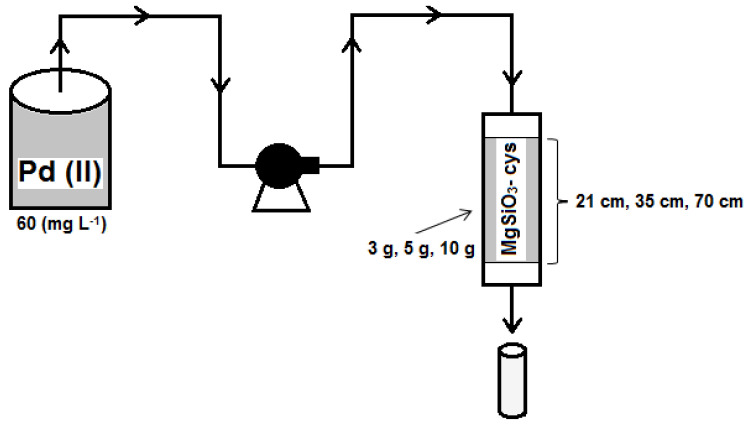
The scheme of the experimental Pd(II) removal installation in a fixed-bed adsorption column.

The experimental setup was made using a glass column (diameter 20 mm and height 300 mm) loaded with three different amounts of adsorbent material (10, 5, and 3 g), corresponding to three layers’ heights (70, 35, and 21 mm, respectively) (Figure 2). The Pd(II) solution was transferred in the experimental column using a peristaltic pump (Heidolph SP quick, Heidolph Instruments, Schwabach, Germany) at a flow rate of 7 (mL min^−1^). The studies were made using samples sequences of 25 mL. The retention times of the solution in the adsorption column, corresponding to the amounts of adsorbent mentioned above, were approximately 3, 1.5, and 1 min, respectively. The residual concentration of Pd(II) was measured using an atomic absorption spectrometer type Varian AAS 280 FS (Varian Inc., Mulgrave, Australia). 

For each adsorption column experiment, there were three independent replicates.

The column adsorption mechanism was established using four specific models: Bohart–Adams, Thomas, Yoon–Nelson, and Clark (linear form), according to equations described elsewhere [60,61,62,63,64,65].

(4) $$\mathrm{Bohart}\text{–}\mathrm{Adams}\;\ln\left(\frac{C_{t}}{C_{0}}\right)=k_{BA}\cdot C_{0}\cdot t-k_{BA}\cdot N_{0}\cdot \frac{Z}{F}$$

(5) $$\mathrm{Thomas}\;\ln\left(\frac{C_{0}}{C_{t}}-1\right)=\frac{k_{Th}\cdot q_{Th}\cdot m}{Q}-k_{Th}\cdot C_{0}\cdot t$$

(6) $$\mathrm{Yoon}\text{–}\mathrm{Nelson}\;\ln\left(\frac{C_{t}}{C_{0}-C_{t}}\right)=k_{YN}\cdot t-\tau \cdot k_{YN}$$

(7) $$\mathrm{Clark}\;\ln\left({\left(\frac{C_{t}}{C_{0}-C_{t}}\right)}^{n-1}\right)=\ln A-r\cdot t$$

where *C*_0_ is the influent concentration (mg L^−1^); *C_t_* is the solution concentration at time *t* in the effluent (mg L^−1^); *t* is time (min); *k_BA_* is the kinetic constant of the Bohart–Adam model (L mg^−1^ min^−1^); *F* is the linear velocity calculated by dividing the flow rate by the column section area; *Z* is the bed depth of column (cm); *N*_0_ is the saturation concentration (mg L^−1^); *k_Th_* is the Thomas rate constant (L min^−1^ mg^−1^); *q_Th_* is the equilibrium compound uptake per g of the resin (mg g^−1^); *m* is the mass of sorbent resin (g); *Q* is the flow rate (mL min^−1^); *k_YN_* is the rate constant (min^−1^); *τ* is the time required for 50% adsorbate breakthrough (min); *n* is the Freundlich constant determined experimentally in batch; *r* is the Clark model constant (min^−1^); and *A* is the Clark model constant.

## 3. Results and Discussion

### 3.1. Characterization of the MgSiO_3_-Cys

BET analysis showed that the specific surface area was S_BET_ = 166 (m^2^ g^−1^). The average pore size and cumulative pore volume calculated using BJH method were 25.24 nm and 0.43 (cm^3^ g^−1^), respectively. The value of pH_PZC_ was six and the density was about 4 (g cm^−3^).

**Figure 3:**
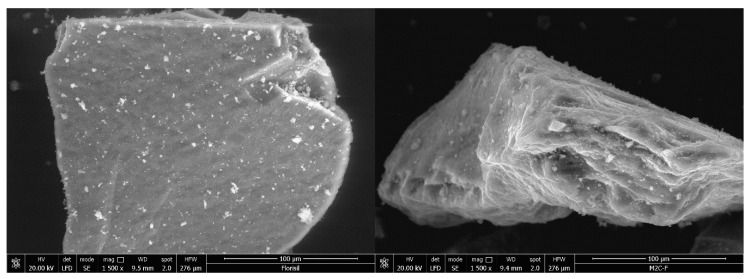
SEM image of adsorbent before and after impregnation.

**Figure 4:**
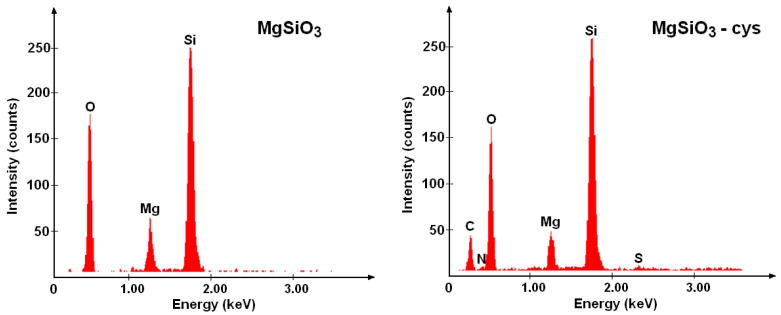
Energy dispersive X-ray (EDX) spectra for MgSiO_3_ and MgSiO_3_-cys materials.

Figure 3 shows the main morphological changes of the surface of the adsorbent material that appeared after impregnation. The presence of functional groups in MgSiO_3_-cys was investigated using energy dispersive X-ray spectroscopy (EDX) (Figure 4). The EDX spectra of MgSiO_3_-cys show both magnesium silicate peaks (O, Mg, Si) and N, S, and C characteristic peaks, confirming the presence of specific peaks for functionalized sorbent.

**Figure 5:**
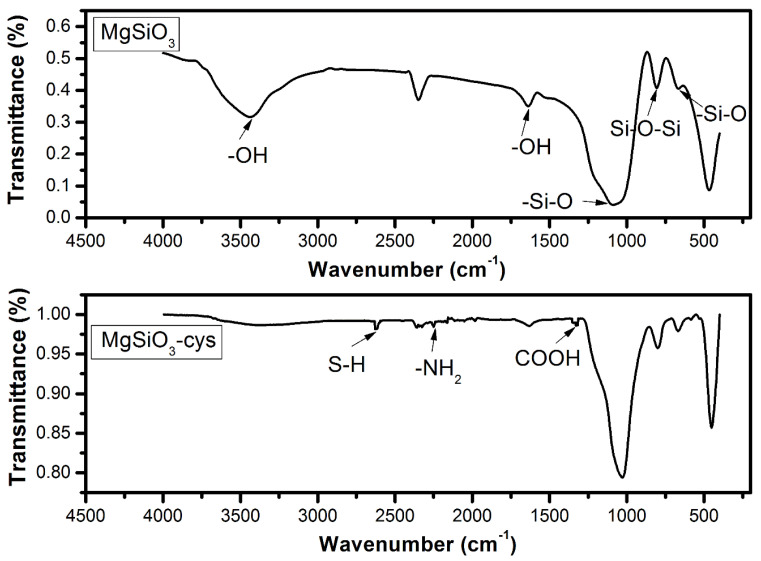
The FTIR spectra for commercial magnesium silicate and functionalized MgSiO_3_-cys.

Infrared spectroscopy (FTIR) was used to confirm the MgSiO_3_ functionalization. The FTIR spectra for commercial magnesium silicate together with functionalized MgSiO_3_-cys are presented in Figure 5. Magnesium silicate-specific peaks can be observed on both spectra: a large peak at 1052 cm^−1^ and another peak at 600 cm^−1^, corresponding to Si–O stretching vibrations, and the peak at 800 cm^−1^, assigned to Si–O–Si bending vibrations. The MgSiO_3_ spectrum show a band at ~3500 cm^−1^ and a peak at 1637 cm^−1^, specific to the –OH bond from H_2_O molecules.

The FTIR spectrum of magnesium silicate functionalized with DL-cysteine shows specific peaks for the –SH bond at ~2620 cm^−1^, the –NH_2_ bond at ~2245 cm^−1^, the –COOH bond at ~1325 cm^−1^. The intensities of those peaks are lower compared to those of MgSiO_3_ due to the small cysteine amount used in the functionalization process. This process leads to an attenuation of MgSiO_3_ specific vibrations.

### 3.2. Equilibrium Adsorption Studies. Adsorption Isotherms

**Figure 6:**
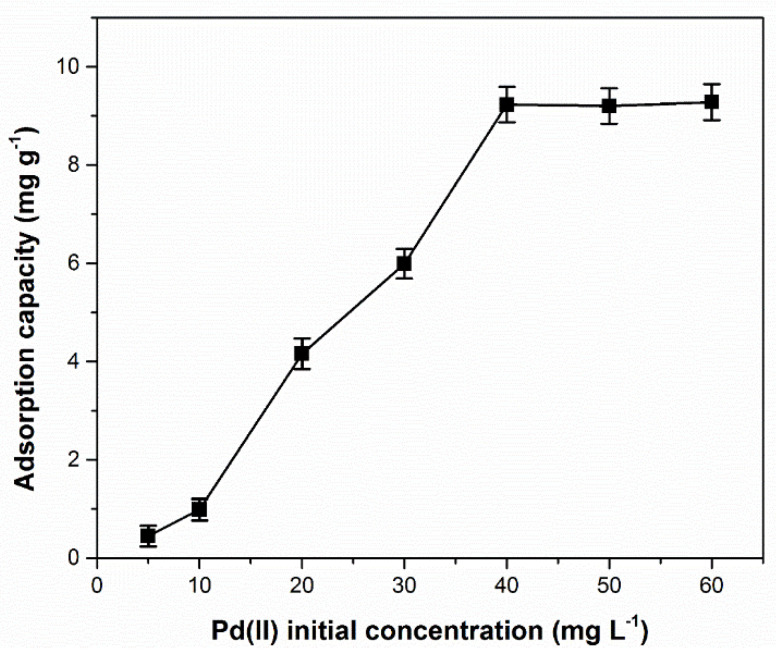
Influence of Pd(II) initial solution concentration on adsorption capacity of MgSiO_3_-cys.

The maximum adsorption capacity of the MgSiO_3_-cys material was determined based on adsorption experiment data using three isotherm models: Langmuir, Freundlich, and Sips [66]. The equilibrium adsorption capacity was determined by monitoring the dependence of materials’ adsorption capacity vs. initial Pd(II) concentration, illustrated in Figure 6.

Augmentation of the initial Pd(II) solution concentration led to an increase in the adsorption capacity up to an approximately constant value. The highest Pd(II) adsorption capacity (*q_m_*) on DL-cysteine functionalized magnesium silicate, for a steady state concentration of 40 (mg L^−1^), was 9.23 (mg g^−1^).

**Figure 7:**
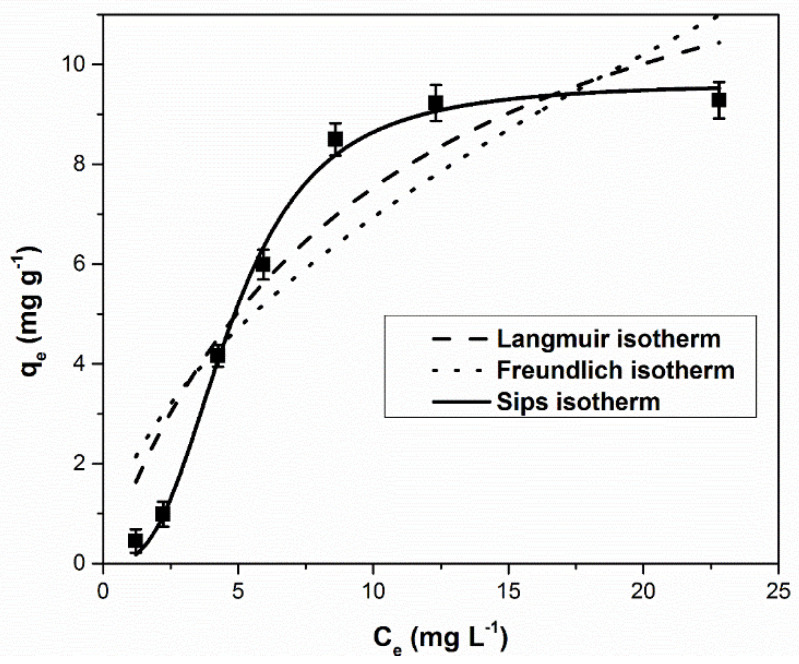
Equilibrium isotherms for adsorption of Pd(II) ions onto MgSiO_3_-cys.

Figure 7 shows the equilibrium isotherms for the studied material. The parameters of the isotherm models for palladium ion adsorption on the studied functionalized material are presented in Table 1.

The existing literature data [67] suggest that most metallic ion adsorption processes on the MgSiO_3_-cys material obtained by chemical modification are multilayer processes and the surface is heterogeneous. At the same time, the adsorption mechanism is controlled by chemisorption processes due to the strong chelation between metal ions and OH^−^ groups or free electron pairs of S and/or N—containing pendant groups present on the surface of the chemical functionalized material.

Using the Sips isotherm to model the obtained experimental data leads to a parameter 1/*n_s_* value deviated from unity, suggesting the heterogeneity of the adsorbent surface [68]. Regardless of the extractant used for functionalization, the Sips model better describes the adsorption process, reflected by the highest correlation coefficient (R^2^) values. In the case of Pd(III) adsorbed on DL-cysteine functionalized magnesium silicate, the correlation coefficient of the Sips isotherm, R^2^ = 0.9953, is higher than those obtained using the Langmuir and Freundlich adsorption isotherms. In addition, the calculated equilibrium adsorption capacity of the Sips model (9.62 mg g^−1^) was consistent with that obtained experimentally (9.23 mg g^−1^).

### 3.3. Bed Height Column (BHC) Influence on the Pd(II) Breakthrough Curves

An important parameter in the sorption process is the bed depth. Pd(II) retention in a fixed-bed column depends, among other factors, on the sorbent quantity reflected by the bed depth of the column works. Three different heights of the MgSiO_3_-cys sorbent filling the fixed-bed column were used in the experiments: 2.1, 3.5, and 7.0 cm.

**Figure 8:**
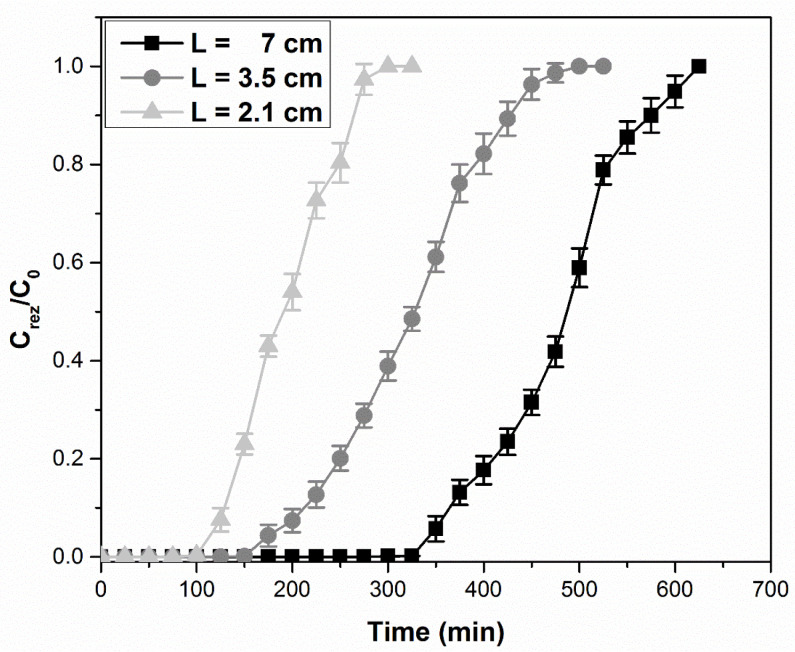
Breakthrough curves for Pd(II) adsorption on a MgSiO_3_-cys fixed-bed column at three fixed-bed bed height columns (BHCs) (T = 298 K, L = 2.1–7.0 cm, Ci = 60 mg L^−1^ Pd(II), and Q = 7 mL min^−1^).

According to Figure 8, the column adsorption process is highlighted by establishing breakthrough curves, which represent the variation of the ratio between the residual concentration of Pd(II) and its initial concentration (*C_rez_*/*C*_0_), depending on the volume of effluent passed through the column, for three distinct amounts of material. Volumes of Pd(II) of 60 (mg L^−1^) concentration were varied between 1500 and 3000 mL, depending on the amount of adsorbent material in the column.

The mass transfer area is the active surface of the bed of adsorbent material where the adsorption of Pd(II) ions takes place [65]. The first part of the column adsorption process takes place rapidly, through the adsorption of Pd(II) on the surface of the material, called the primary adsorption zone. This is why, at the beginning, the collected samples do not contain Pd(II) ions. The second part of the adsorption process is slower and is characterized by the adsorption of Pd(II) ions on the adsorbent material, achieving mass transfer. The adsorption process is complete, the concentration of Pd(II) ions varies from 60 to 0 (mg L^−1^), and the saturation of the material is total.

For the three different bed depths used, as the bed depth increases (from 2.1 at 7 cm), the breakthrough point increases (from 100 to 325 min). A rational explanation of this behavior is that with increasing column sorbent height a greater number of binding sites become available and the quantity of the Pd(II) removed increases accordingly [69].

A higher BHC leads to a longer contact time between the waste solution and the MgSiO_3_-cys sorbent (from about 1 min to 3 min), having a positive influence on Pd(II) adsorption.

Figure 8 shows an alteration of the steep concave shape to flat concave shape curves as BHC increases, which leads to an enlargement of the mass transfer area [70,71,72]. However, too high a layer of adsorbent material in the column is not recommended as it increases the flow resistance [73].

### 3.4. Modeling for Adsorption Behaviors of Pd(II) on MgSiO_3_-Cys

Various practical parameters such as sorbent capacity, contact time between adsorbent and adsorbed, column operating life span, regeneration time, and prediction of the time necessarily have a significant influence upon the operation of the column. Knowing these parameters is important to model the adsorption process in a fixed-bed column.

The four models tested (Bohart–Adams, Yoon–Nelson, Thomas, and Clark) provide detailed conclusions about the process mechanism. The adsorption column is subjected to axial dispersion, external film strength, and intraparticle diffusion resistance [74].

The Bohart–Adams model is used for one-component systems and provides information on the saturation concentration of the material. This model characterizes the beginning of the column penetration, gives information about the adsorbent material used, and shows the maximum concentration at which the column is instantly broken through [60]. The Yoon–Nelson model is used to model a one-component system and provides information about the time by which half of the column is broken through. It is a purely theoretical model which does not focus on the properties of the adsorbent, the type of adsorbent, or the physical characteristics of the fixed bed [74]. The Thomas model provides information on the maximum solid phase concentration of the adsorbent and on the rate constant [62], while the Clark model describes, very well, the dynamic adsorption process [74].

#### 3.4.1. Bohart–Adams Model

**Figure 9:**
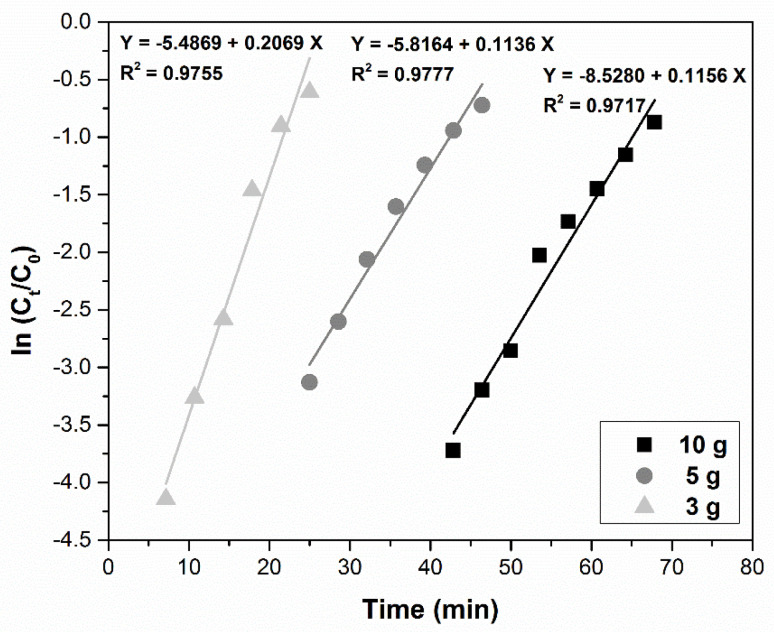
Bohart–Adams model for the adsorption of Pd(II) in a fixed-bed column at various MgSiO_3_-cys amounts.

Figure 9 illustrates the influence of sorbent dose (10, 5, and 3 g) corresponding to the three previously mentioned BHCs (7, 3.5, and 2.1 cm) on the ln (*C_t_*/*C*_0_) vs. time curves. The graphic shows a direct influence of the sorbent amount upon maximum adsorption capacity, *N*_0_, and the kinetic constant, *k_BA_*, which indicates that, kinetically, the process is controlled by the mass transfer in the first part of the breakthrough process. The calculated regression coefficients have relatively low values (between 0.9717 and 0.9755); therefore, one can assume that the model is not the most suitable to describe the Pd(II) adsorption mechanism on MgSiO_3_-cys in a dynamic regime.

#### 3.4.2. Thomas Model

**Figure 10:**
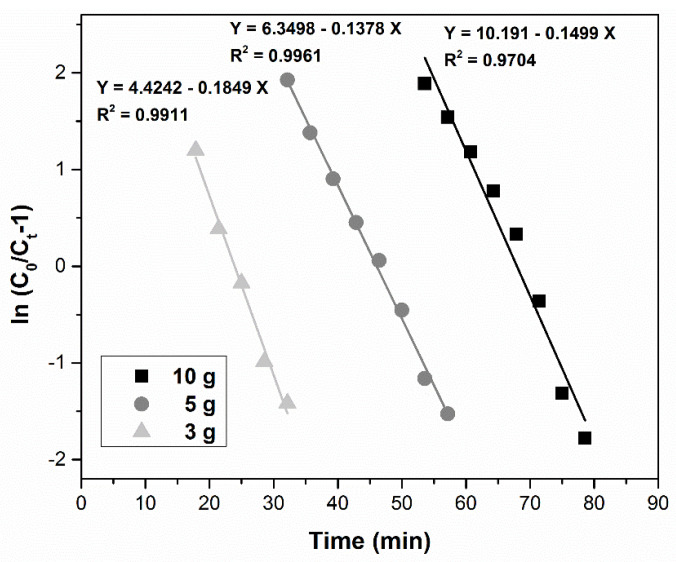
Thomas model for the adsorption of Pd(II) in a fixed-bed column at various MgSiO_3_-cys amounts.

Figure 10 shows the influence of sorbent dose (10, 5, and 3 g) upon the ln[(*C*_0_*/C_t_*) − 1] vs. time curves. The figure illustrates that a higher sorbent amount leads to a decrease in the Thomas rate constant *k_Th_*. The reason for this behavior is the adsorption driving force given by the difference between the Pd(II) concentration in the sorbent and in the solution [75,76,77,78]. The determination coefficient R^2^ values (between 0.9704 and 0.9961) indicated positive correlation, but we cannot assume that this model is best fitted for the adsorption mechanism. The adsorption capacity *q_Th_* and the kinetic constant are presented in Table 2.

#### 3.4.3. Yoon–Nelson Model

**Figure 11:**
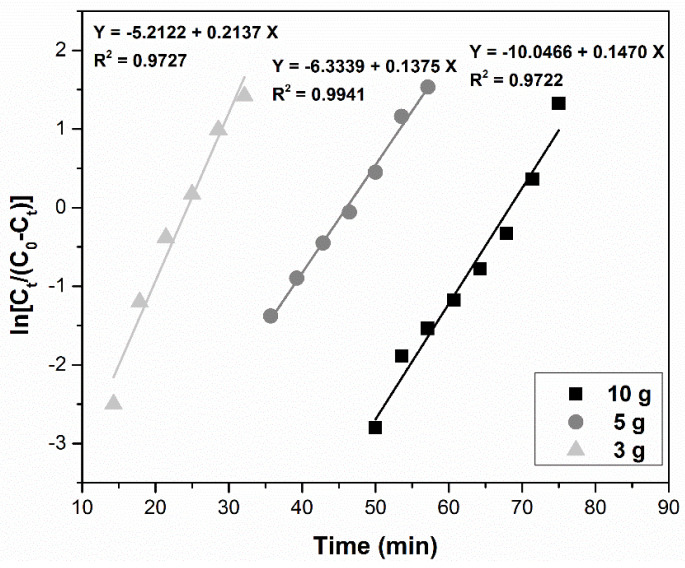
Yoon–Nelson model for the adsorption of Pd(II) in a fixed-bed column at various MgSiO_3_-cys amounts.

Figure 11 illustrates the relationship of ln[*C_t_*/(*C*_0_ − *C_t_*)] vs. time for the three sorbent doses. Increasing the sorbent mass leads to an increase in the breakthrough time and also in the *k_YN_* constant, as it is presented in Table 2. The determination coefficient R^2^ ranged between 0.9722 and 0.9941 but we cannot assume that it best describes the adsorption process.

#### 3.4.4. Clark Model

**Figure 12:**
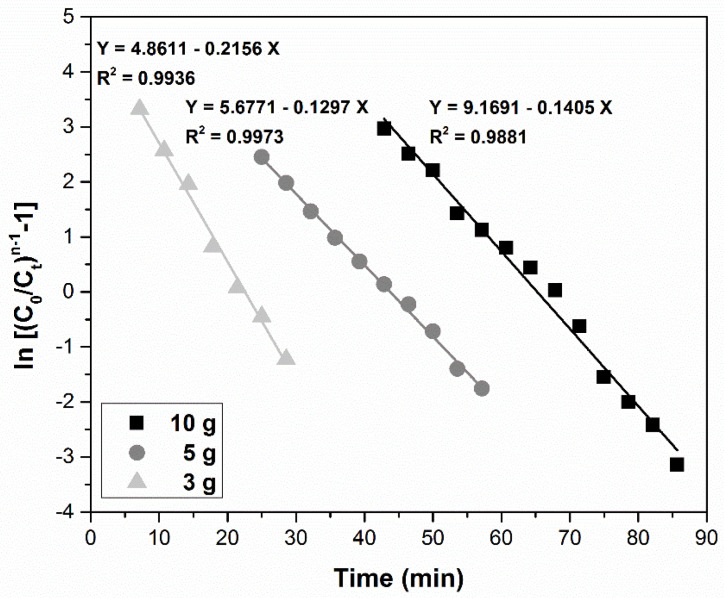
Clark model for the adsorption of Pd(II) in a fixed-bed column at various MgSiO_3_-cys amounts.

The relationship ln[(*C*_0_*/C_t_)^n^*^−1^ − 1] vs. time for all adsorbent doses studied is shown in Figure 12, where *n* is the Freundlich constant, determined experimentally in batch section. The value of this parameter was 1.81. The high values of the determination coefficient R^2^ (between 0.9881 and 0.9973) certify that the Clark model best describes the adsorption in a fixed-bed column. The value of *r* and *A* parameters are presented in Table 2.

Table 3 provides a comparison between the DL-cysteine functionalized magnesium silicate obtained in this study and other commonly used sorbents for Pd(II) removal in batch systems. The equilibrium adsorption capacity value is comparable to or even higher than those obtained in previous studies using various adsorbent materials. This behavior is based on the presence of the -SH and -NH_2_ groups in functionalized material structures, suggesting the surface adsorption of Pd(II) by free electrons or by creating hydrogen bridges.

In addition, the experimental conditions in which the adsorption studies were performed, for each material, are highlighted.

## 4. Conclusions

The current paper presents a new adsorbent, MgSiO_3_ functionalized with DL-cysteine (cys), designed for palladium ion recovery from waste solutions.

SEM, EDX and FTIR analyses revealed morphological changes in the surface of the adsorbent material after impregnation and confirmed the functionalization of MgSiO_3_ with DL-cysteine.

The modeling of the experimental data obtained in the batch system showed that the Sips isotherm best describes the adsorption process, because the correlation coefficient R^2^ approaches 1 and the maximum calculated adsorption capacity (9.62 mg g^−1^) is close to the experimentally determined value (9.23 mg g^−1^). The obtained adsorption capacity is better than those reported in the literature for other adsorbent materials due to the presence of the -SH and -NH_2_ groups in the structure of the functionalized material which allow the surface adsorption of Pd(II) by free electrons or by creating hydrogen bridges.

Palladium ion adsorption studies in a dynamic regime using a fixed-bed column are influenced by the adsorbent bed height (the output flow rate decreases as the fixed-bed height increases). The adsorption process is characterized by the Clark model for all the MgSiO_3_-cys material bed heights studied.

## Figures and Tables

**Table 1:** Parameters of isotherm models for adsorption of palladium ions onto MgSiO_3_-cys.

*q_m,exp_* (mg g^−1^)	Freundlich Isotherm			Langmuir Isotherm			Sips Isotherm			
	*K_F_* (mg g^−1^)	1/*n*	R^2^	*K_L_* (L mg^−1^)	*q_m_* (mg g^−1^)	R^2^	*K_S_*	*q_m_* (mg g^−1^)	1/*n_S_*	R^2^
9.23	1.92	0.55	0.7599	0.10	14.95	0.8688	0.01	9.62	0.34	0.9953

**Table 2:** Pd(II) adsorption process parameters in a fixed-bed column.

Column Adsorption Parameters Specification				
Bohart–Adams model	MgSiO_3_-cys amounts (g)	*k_BA_* (L mg^−1^ min^−1^)	*N*_0_ (mg L^−1^)	R^2^
	3	3.45 × 10^−3^	1688.8	0.9755
	5	1.89 × 10^−3^	1960.8	0.9777
	10	1.90 × 10^−3^	1407.7	0.9717
Thomas model	MgSiO_3_-cys amounts (g)	*k_Th_* (L mg^−1^ min^−1^)	*q_Th_* (mg g^−1^)	R^2^
	3	2.50 × 10^−3^	3.33	0.9911
	5	2.29 × 10^−3^	3.88	0.9961
	10	2.99 × 10^−3^	2.85	0.9704
Yoon–Nelson model	MgSiO_3_-cys amounts (g)	*k_YN_* (min^−1^)	*τ* (min)	R^2^
	3	0.2137	24.40	0.9727
	5	0.1376	46.05	0.9941
	10	0.1469	68.30	0.9722
Clark model	MgSiO_3_-cys amounts (g)	*r* (min^−1^)	*A*	R^2^
	3	0.2156	9509	0.9936
	5	0.1297	290	0.9973
	10	0.1405	129	0.9881

**Table 3:** The equilibrium adsorption capacity of different sorbents reported in literature compared to the proposed MgSiO_3_-cys material.

Material	Adsorption Conditions	Adsorption Capacity, (mg g^−1^)	References
Lewatit MP-500 resin	Contact time = 2 h Temperature = 298 K Initial concentration of Pd(II) complex = 0.1 M	8.45	[79]
Nonylthiourea-coated Fe_3_O_4_	Contact time = 30 min Temperature = 295 K pH = 2.5 Initial concentration of Pd(II) = 0.076 (mmol g^−1^)	8.10	[80]
Grape stalk impregnated with orthophosphoric acid	Contact time = 4 h Temperature = 295 K pH = 1.5 Initial concentration of Pd(II) = 60 (mg L^−1^)	1.4	[81]
Thiocyanate retaining tannin gel	Contact time = 2 h, Temperature = 298 K, Initial concentration of Pd(II) = 0.001 M	0.065	[82]
Crosslinked carboxymethyl chitosan hydrogels	Contact time = 2 h Temperature = 295 K pH = 4, Initial concentration of Pd(II) = 100 ppb	2.6	[83]
Fungi *Aspergillus* sp.	Contact time = 45 min, Temperature = 298 K pH = 2.5–3.5 Initial concentration of Pd(II) = 0.075 (mg L^−1^)	4.28	[84]
MgSiO_3_-cys	Contact time = 1 h Temperature = 298 K pH = 2 Initial concentration of Pd(II) = 40 (mg L^−1^)	9.23	This paper
